# Diagnosing microbial keratitis in different settings

**Published:** 2025-01-31

**Authors:** Astrid Leck, Reena Yadav, Abel Ebong, Simon Arunga, Jeremy Hoffman

**Affiliations:** 1 Assistant Professor: International Centre for Eye Health, LSHTM, London, UK.; 2 Consultant Ophthalmologist and Head of Department, Cornea: Sagarmatha Choudhary Eye Hospital (SCEH), Lahan, Nepal.; 3 Ophthalmologist: Mbarara University of Science and Technology, Mbarara, Uganda.; 4 Senior Lecturer, Department of Ophthalmology: Mbarara University of Science and Technology and Honorary Assistant Professor: International Centre for Eye Health, LSHTM, UK.; 5 Consultant Ophthalmologist and Corneal Service Lead: Buckinghamshire Healthcare NHS Trust and Clinical Research Fellow: International Centre for Eye Health, LSHTM, UK.

**Microbial keratitis is an ocular emergency. Identifying the likely cause can help to save the eye and preserve vision.**

Microbial keratitis refers to severe corneal infection caused by a variety of microorganisms, including bacteria, fungi, viruses, and protozoa, such as *Acanthamoeba*; treatment depends on the type of microorganism involved.

Patients with microbial keratitis all present with a red, painful and light-sensitive eye. There may have been a history of trauma or contact lens wear. Clinical signs of microbial keratitis include a red eye with a white patch or infiltrate over the cornea. If fluorescein staining is available, the ulcer or epithelial defect will fluoresce green under a blue light. These signs are similar, regardless of the type of microorganism responsible, which makes diagnosis, and therefore management, a challenge. In an ideal setting, formal diagnosis is made following corneal scrapes for microscopy and culture. However, this is not always available, particularly in rural or low-resource settings.

It is therefore important to try to determine the type of infection with the resources that you have available, in order to give the patient the best possible outcome.

## Epidemiology

Knowing which causes of microbial keratitis are common where you work is helpful if you need to diagnose and treat patients without the support of a diagnostic laboratory.

The incidence of microbial keratitis varies globally, influenced by factors such as occupational risk factors, climate, socioeconomic status, and other risk factors. In rural and low-resource settings, fungal keratitis following agriculture-related ocular trauma is common. In high-income, urban settings, contact lens wear-associated bacterial keratitis may be more prevalent; these would also be the only settings in which you are likely to see patients presenting with *Acanthamoeba* keratitis.

## Diagnosis

### Where there is no ophthalmic expertise or laboratory support

In the community, or at primary health care units where there is no ophthalmic expertise or diagnostic laboratory support, patients with suspected corneal infection should be prescribed broad-spectrum antibiotic eye drops and referred to an eye hospital urgently.

One of the challenges in getting patients the correct treatment, in time, is lack of awareness of the signs of corneal infection.

We therefore encourage you to share the poster on pages 8–9 with pharmacists and health workers in your local area. You may also wish to adapt the community education poster on page 16, which was used to educate farmers in Nepal about the importance of visiting an eye centre if something entered their eye.

### Where there is ophthalmic expertise but no laboratory support

At health centres or hospitals where there is no laboratory support, but there is a trained eye care worker, optometrist, or ophthalmologist, it may be possible to differentiate between fungal and bacterial infection using a diagnostic algorithm based on clinical signs.

Although it is difficult to distinguish bacterial from fungal keratitis on clinical examination alone, there are certain findings that can be suggestive of fungal infection, specifically if the margins have serrated or jagged edges (Figure 1a) and if the ulcer has a raised (or three-dimensional) profile (Figure 1c).^1–3^

**Figure 1:**
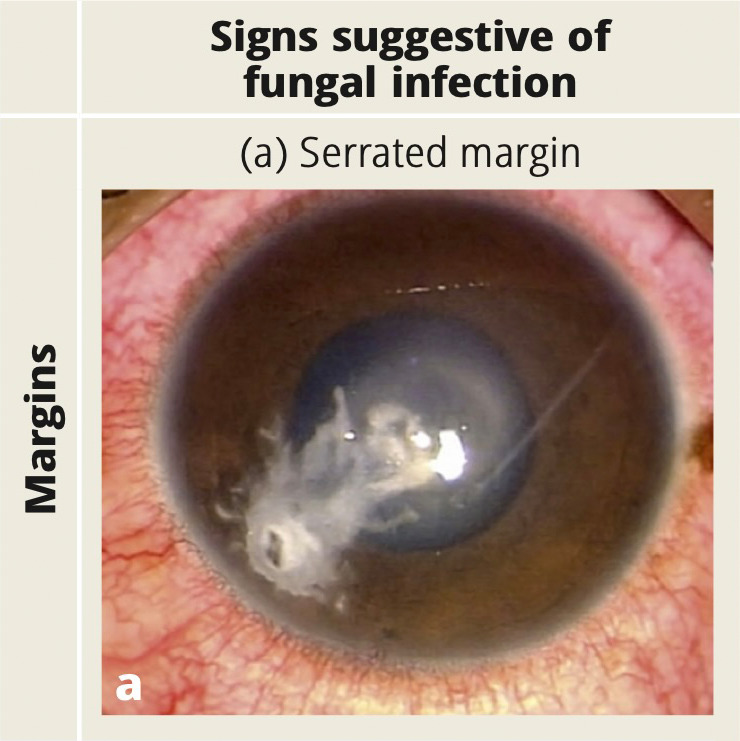

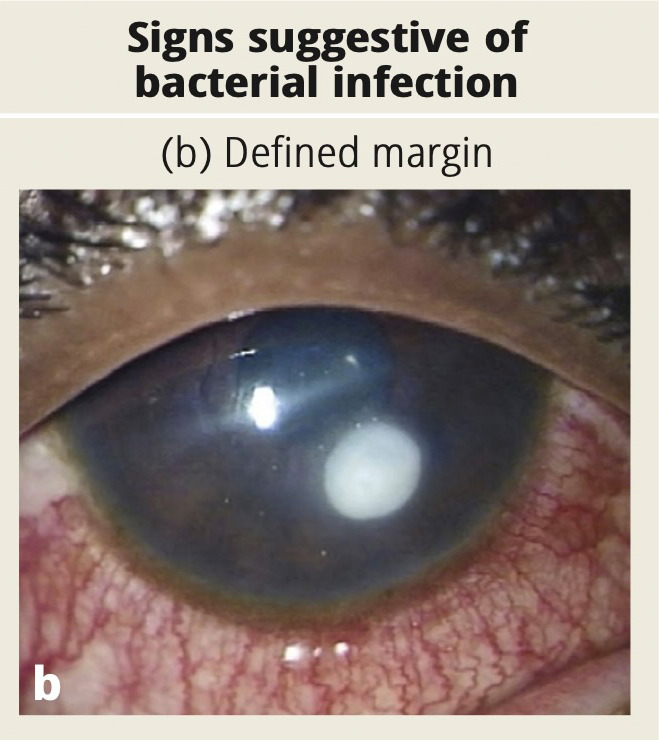

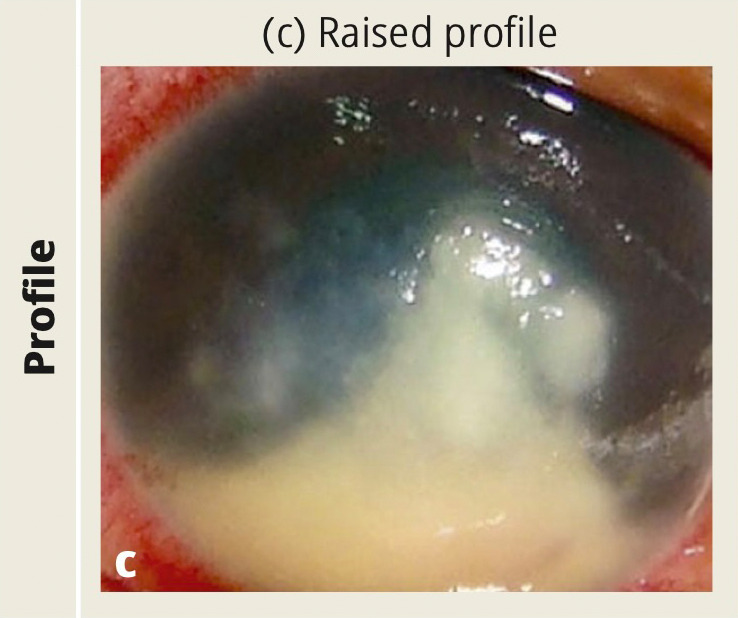

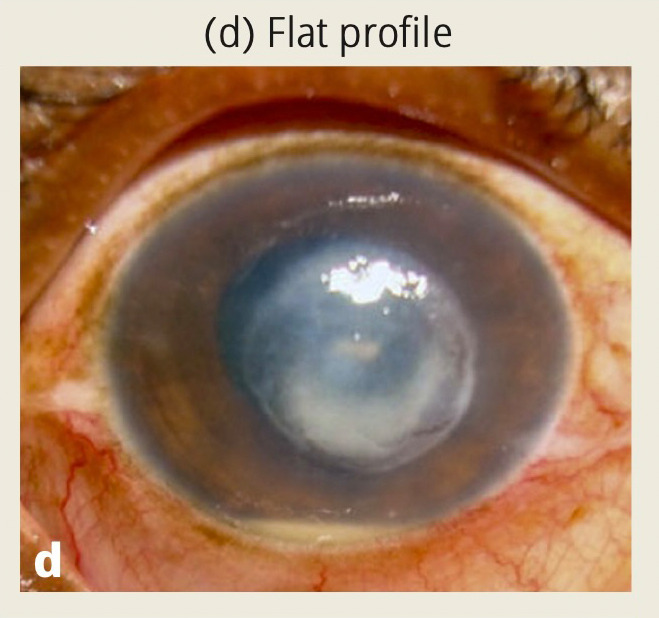
Clinical features typical of fungal (a and c) and bacterial (b and d) infection.

© PHILIP THOMNAS CC BY-NC-SA 4.0

© PHILIP THOMNAS CC BY-NC-SA 4.0

© PHILIP THOMNAS CC BY-NC-SA 4.0

© PHILIP THOMNAS CC BY-NC-SA 4.0

If you are able to examine the anterior chamber, preferably with a slit lamp (to see if there is fibrin) you can use the recommended diagnostic algorithm in Figure 2 to differentiate between bacterial and fungal corneal infection. See Figure 3 for a photo of fibrin in the anterior chamber.

**Figure 2:**
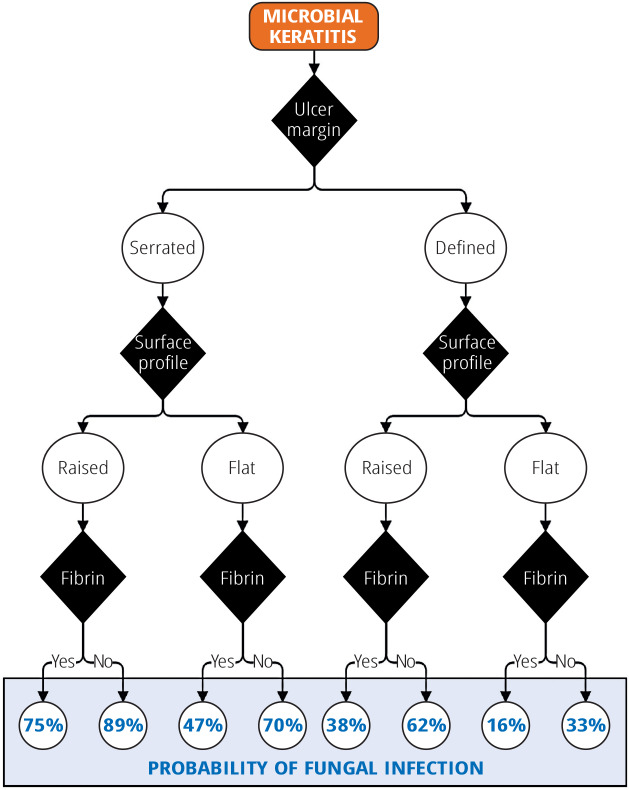
Algorithm for determining the probability of fungal keratitis. The black diamonds are decision points about three clinical features: ulcer or infiltrate margin, surface profile, and anterior chamber fibrin (see Figure 3). These probabilities are based on data presented in Thomas et al.^1^

**Figure 3:**
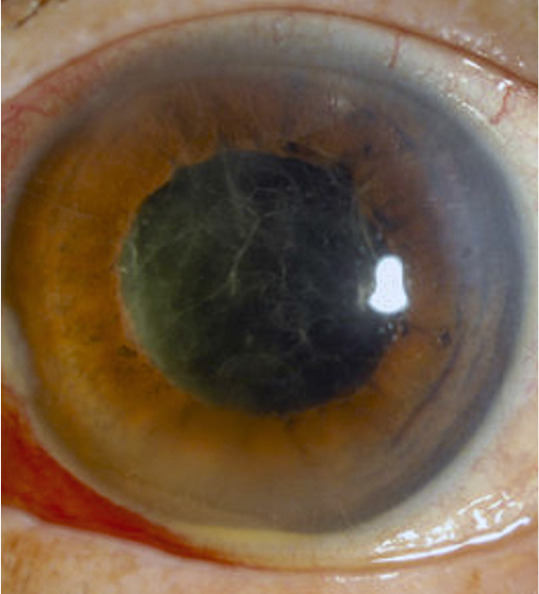
Fibrin seen in the anterior chamber using an ophthalmoscope.

© ICEH CC BY-NC-SA 4.0

### Where there are limited laboratory diagnostics

Microscopy provides a rapid presumptive diagnosis, even in the absence of culture facilities or the absence of microbial growth in culture.

In health care facilities with access to a diagnostic laboratory microscope and a health care professional who is competent in taking a corneal scrape specimen, corneal tissue can be examined using Gram stain and KOH reagents for the presence of bacteria, fungal hyphae, yeast cells, and *Acanthamoeba*. Details on how to perform a corneal scrape can be found here: www.cehjournal.org/articles/583

If microscopic examination of the smear reveals heavy bacterial infection (>30 cells per field of view) the clinician should be advised to begin antimicrobial therapy. If there are moderate numbers of Gram-negative bacilli observed (10–25 bacterial cells), this may also be significant.

If the health centre/hospital has access to a UV microscope, as used in the laboratory diagnosis of tuberculosis (TB), it is possible to use a fluorescent stain to visualise fungal hyphae (Figure 4) and *Acanthamoeba* cysts.

**Figure 4:**
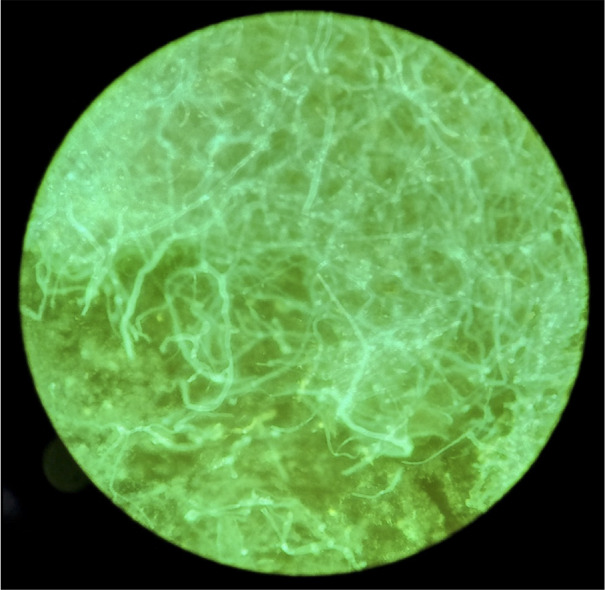
Fungal hyphae in corneal tissue stained with calcofluor white, a fluorescent stain, seen using a UV microscope.

© ISONIA YADAV, SCEH, NEPAL CC BY-NC-SA 4.0

**Note:** The presence of fungal hyphae in corneal tissue is always significant and the patient should be prescribed antifungal eye drops, even in the absence of culture results.

### Where there is a full diagnostic laboratory service, including culture facilities

Tertiary-level centres should perform culture from corneal scrape material; this can confirm the type of infection and identify the causative microorganism, which helps to guide appropriate treatment. If culture is possible, antimicrobial susceptibility testing can also be performed to further inform antimicrobial prescribing and monitor antimicrobial drug resistance profiles.

To definitively confirm bacterial keratitis, the following criteria are applied:

- Growth of the same bacteria is demonstrated on two or more solid phase culture media
- There is semiconfluent bacterial growth at the site of inoculation (C-streak, Figure 5) or growth on one solid medium consistent with microscopy (that is, appropriate staining and morphology with Gram stain)
- Semi-confluent bacterial growth at the site of inoculation on one solid medium (if bacteria) or growth of the same organism on repeated scraping.

**Figure 5:**
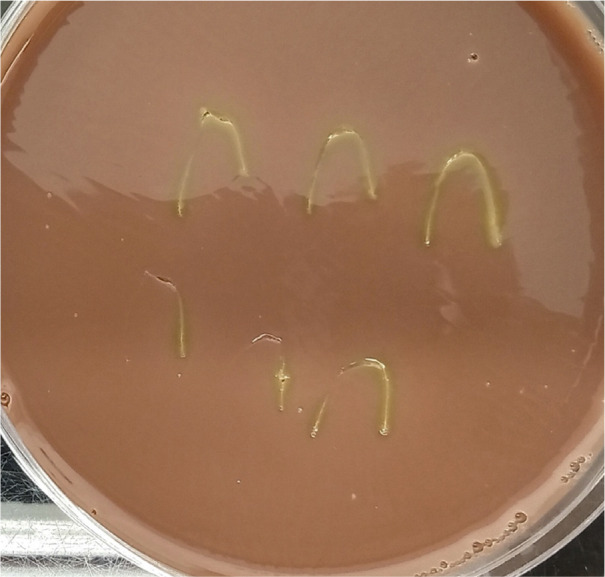
Bacterial growth on C-streaks on chocolate (heated blood) agar.

© ISONIA YADAV, SCEH, NEPAL CC BY-NC-SA 4.0

If culture results are negative, but microscopic examination of the smear reveals heavy bacterial infection (>30 cells per field of view, see Figure 6), the clinician should begin antimicrobial therapy.

**Figure 6:**
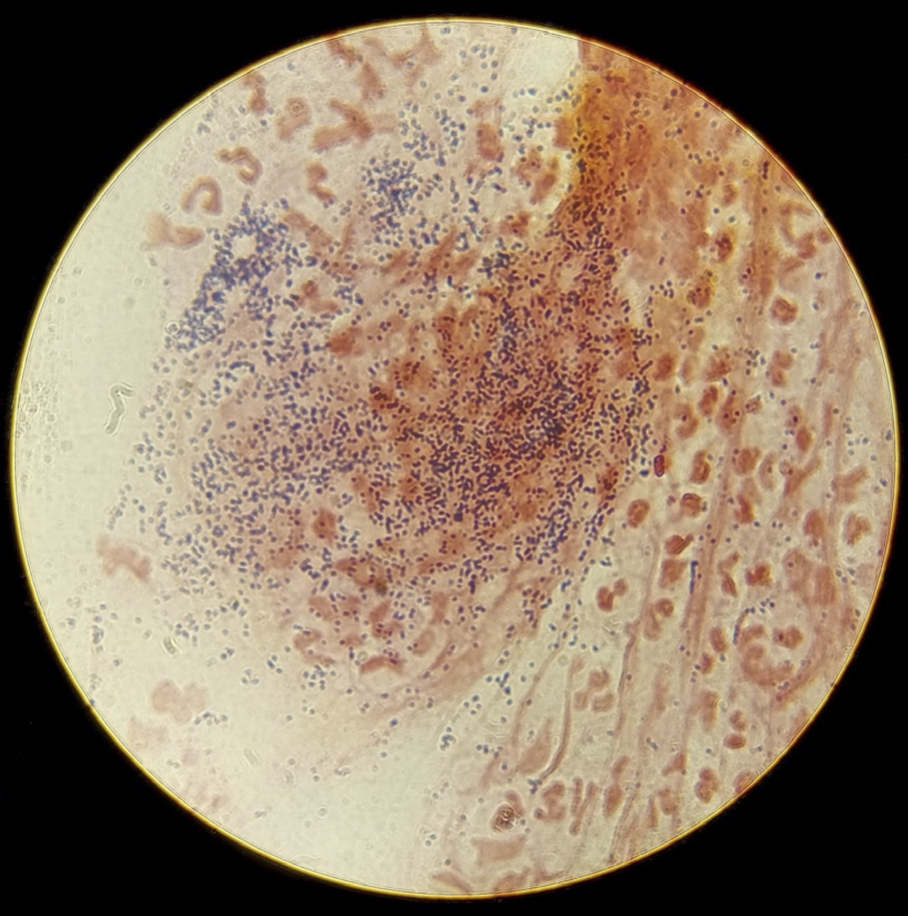
Gram-stained corneal tissue showing a heavy inoculum of Gram-positive cocci (purple dots).

© ISONIA YADAV, SCEH, NEPAL CC BY-NC-SA 4.0

If fungal hyphae are seen in any stained corneal material using light or UV microscopy, this is a definitive diagnosis for fungal infection. If microscopy is negative but there is fungal growth only at the site of C-streak inoculation on one or more solid culture media (agar plate or slope), the causative organism is reported as fungal.

If *Acanthamoeba* are suspected as the cause of an infection, a corneal tissue specimen is taken for microscopic examination. A second corneal tissue specimen is inoculated onto a non-nutrient agar (NNA) plate, a portion of which is transferred in the laboratory to a second non-nutrient agar plate overlaid with *E.coli* to determine viability (Figure 7).

**Figure 7:**
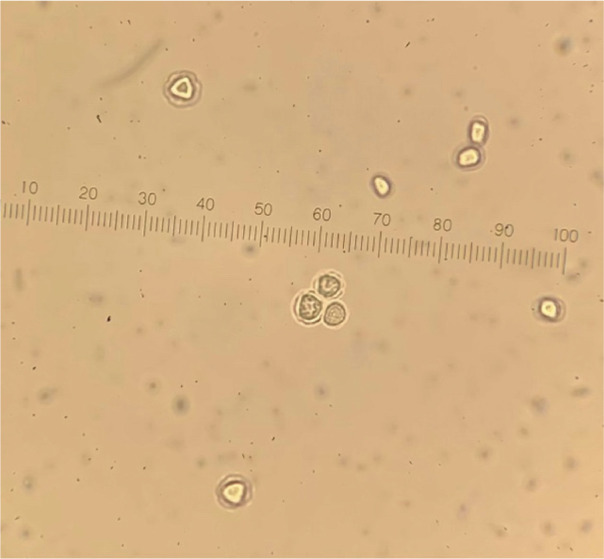
*Acanthamoeba* cysts – wet prep.

© ASTRID LECK CC BY-NC-SA 4.0

“It is important to consider fungal infection until proven otherwise in settings where fungal infection is prevalent

### Key points:

- It is important to consider fungal infection until proven otherwise in settings where fungal infection is prevalent.
- Microscopy is a very sensitive diagnostic tool and can help you to reach a definitive diagnosis for fungal infection.
- Microbial culture is important, particularly for the diagnosis of bacterial infection, and it also enables identification and antimicrobial susceptibility testing to be carried out, increasing our knowledge base of what causes corneal infection within a region and guiding appropriate treatment.

**Types of microbial keratitis**

**Bacterial keratitis**

The most common type of corneal infection worldwide is bacterial keratitis, associated with ocular trauma, contact lens wear, or pre-existing ocular surface disease. Common causative microorganisms include *Staphylococcus aureus*, *Pseudomonas aeruginosa* and *Streptococcus pneumoniae*. Bacterial keratitis typically presents with rapid onset of pain, redness, and tearing.

**Figure 1:**
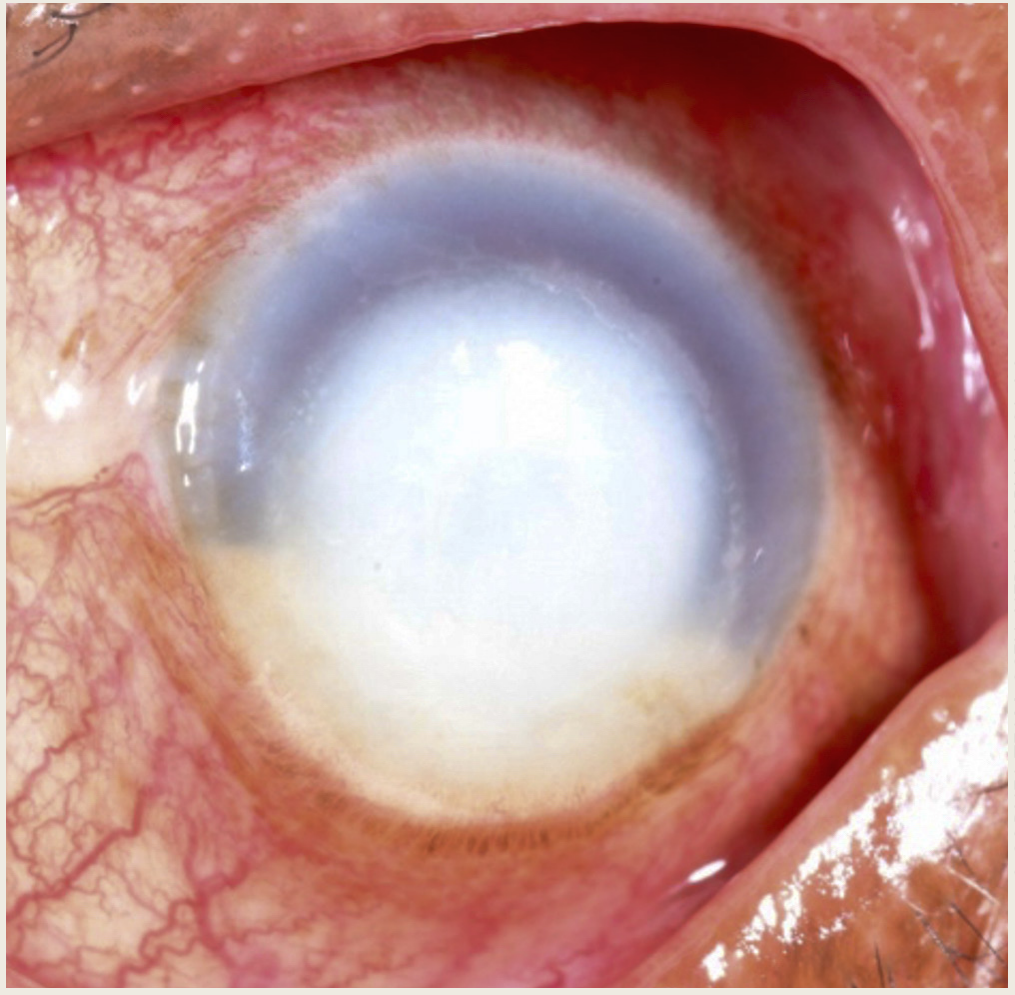
Corneal infection caused by *S.pneumoniae*

© CORNEA TEAM, SCEH, NEPAL CC BY-NC-SA 4.0

**Figure 2:**
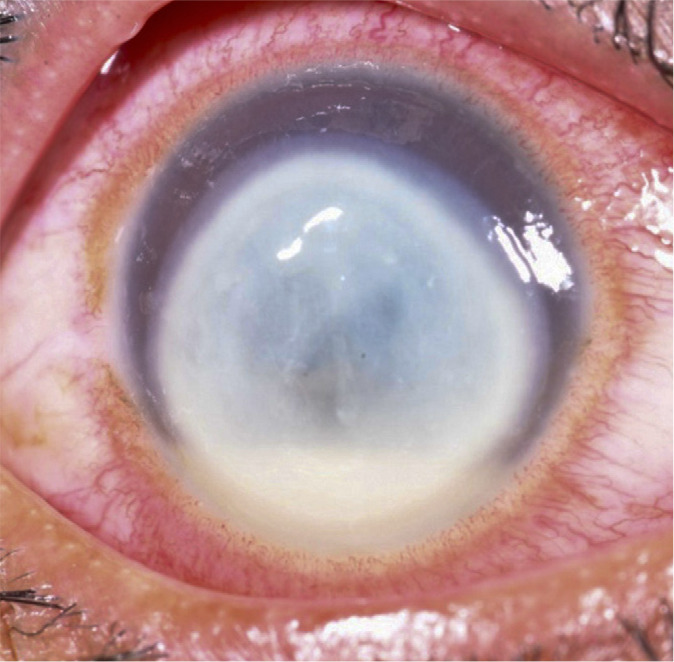
Corneal infection with *P. aeruginosa*

© CORNEA TEAM, SCEH, NEPAL CC BY-NC-SA 4.0

**Figure 3:**
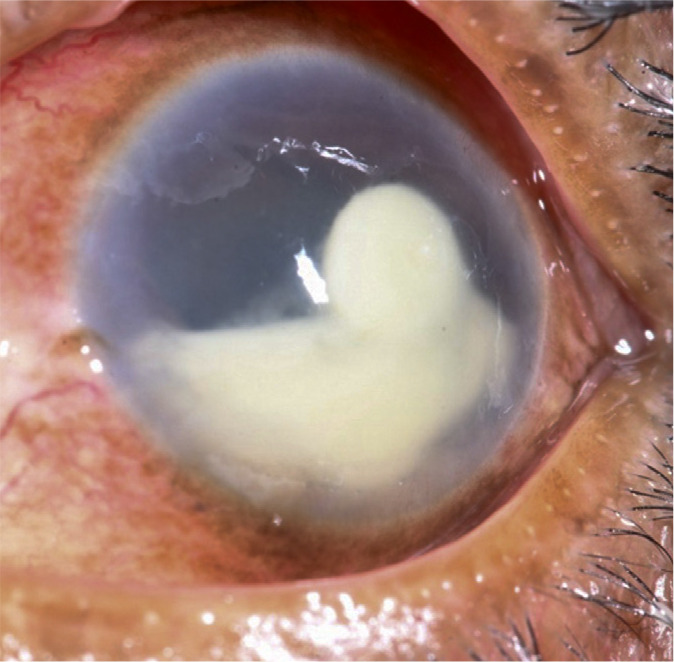
A cornea infected with *S. aureus*

© CORNEA TEAM, SCEH, NEPAL CC BY-NC-SA 4.0

**Fungal keratitis**

Predominantly seen in tropical and subtropical climatic regions, fungal keratitis is frequently associated with agriculture-related ocular trauma involving plant material. Pathogens such as *Fusarium*, *Aspergillus* and *Curvularia* species are frequently responsible. This type of infection often presents with a slower onset and more drawn-out course compared to bacterial keratitis, but the clinical signs can be similar similar to bacterial keratitis.

**Figure 4:**
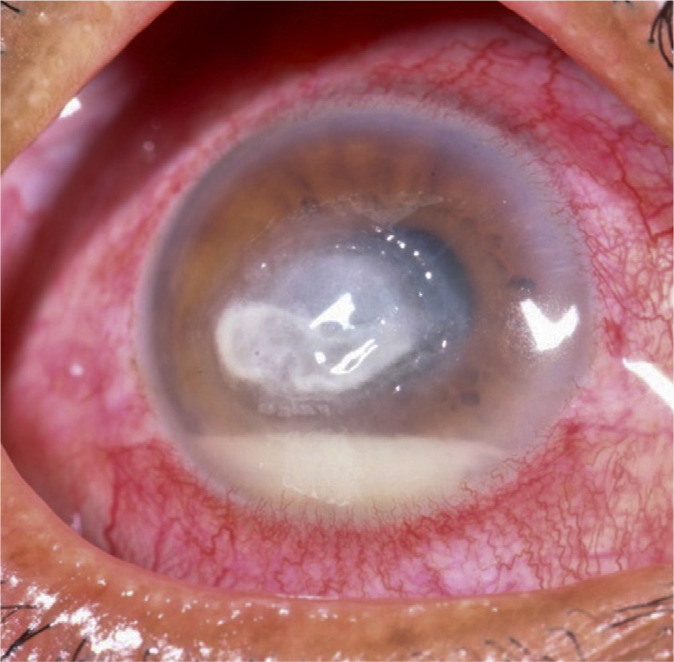
*Fusarium* keratitis

© CORNEA TEAM, SCEH, NEPAL CC BY-NC-SA 4.0

**Figure 5:**
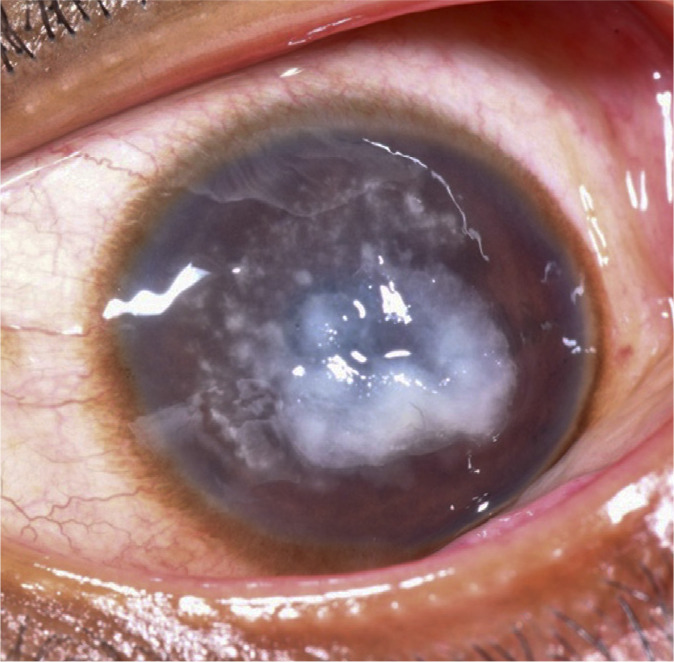
Corneal infection due to *Aspergillus sp*.

© CORNEA TEAM, SCEH, NEPAL CC BY-NC-SA 4.0

**Figure 6:**
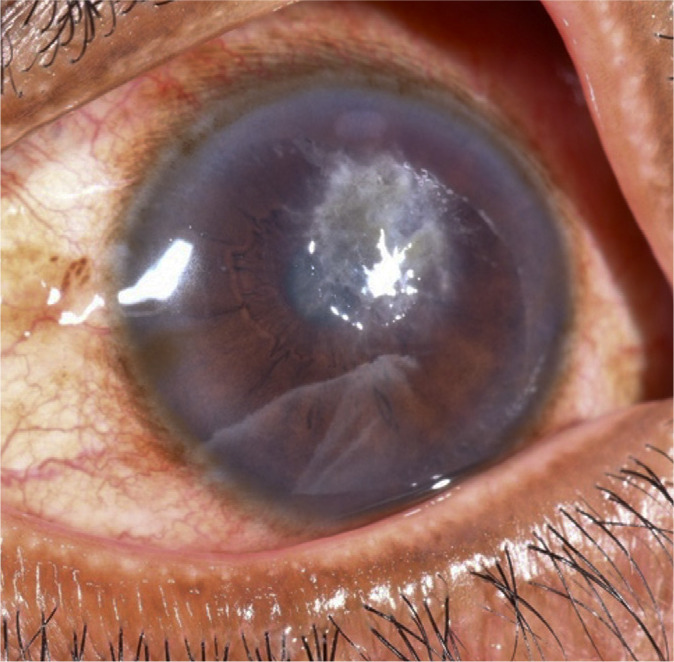
*Curvularia* keratitis

© CORNEA TEAM, SCEH, NEPAL CC BY-NC-SA 4.0

**Viral keratitis**

Primarily caused by herpes simplex virus (HSV) and, less frequently, by varicella-zoster virus (VZV), viral keratitis can lead to recurrent episodes and chronic complications. Herpetic keratitis is characterised by dendritic or geographic ulcers, stromal keratitis, and potential for chronic scarring. Viral keratitis can be diagnosed using blue light examination at a slit lamp – no laboratory investigations are needed.

**Figure 7:**
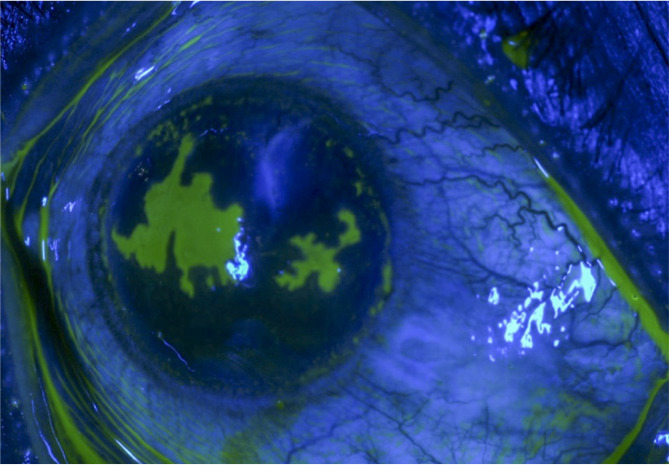
Dendritic appearance of a viral corneal infection

© CORNEA TEAM, SCEH, NEPAL CC BY-NC-SA 4.0

**Figure 8:**
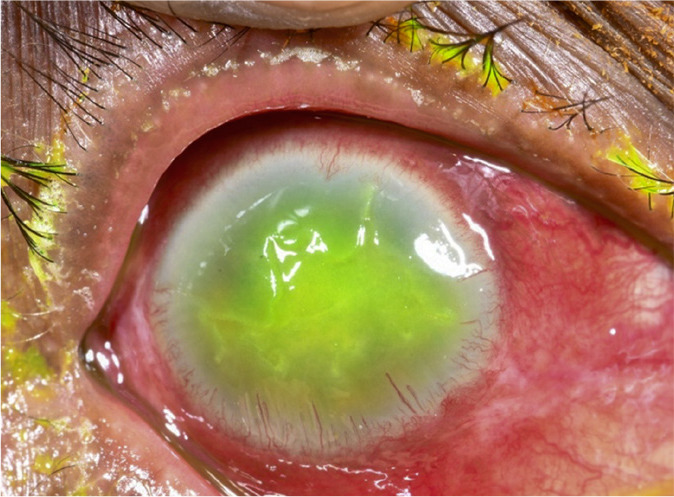
Geographical ulcer (viral)

© CORNEA TEAM, SCEH, NEPAL CC BY-NC-SA 4.0

***Acanthamoeba* keratitis**

Although less common, keratitis caused by *Acanthamoeba* spp.is particularly severe and challenging to treat. It is typically associated with **improper contact lens hygiene,** or **exposure to fresh water sources containing the protozoa**. In early disease, patients can present with non-specific signs, but at a later stage they can develop severe pain due to the infiltration of corneal nerves.

**Figure 9:**
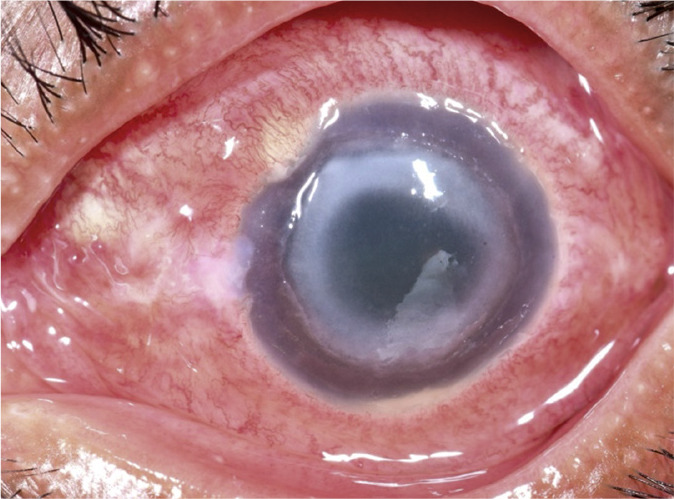
*Acanthamoeba* keratitis

© CORNEA TEAM, SCEH, NEPAL CC BY-NC-SA 4.0
